# Tacrolimus-Induced Acute Esophageal Necrosis Postorthotopic Liver Transplantation

**DOI:** 10.14309/crj.0000000000001772

**Published:** 2025-07-18

**Authors:** Wei Tang, Makeda Dawkins, Daniel Basta, Nao Hara, Fouzia Shakil, Bodin Roxana, Shireen Pais, Kenji Okumura, Seigo Nishida, Abhay Dhand

**Affiliations:** 1 Division of General Internal Medicine, Westchester Medical Center, Valhalla, NY; 2 Division of Gastroenterology and Hepatobiliary Diseases, Westchester Medical Center, Valhalla, NY; 3 Department of Pathology, Westchester Medical Center, Valhalla, NY; 4 Division of Gastroenterology and Hepatobiliary Diseases, Advanced Endoscopy, Westchester Medical Center, Valhalla, NY; 5 Division of Transplant Surgery, Westchester Medical Center, Valhalla, NY; 6 Transplant Infectious Diseases, Westchester Medical Center, Valhalla, NY

**Keywords:** acute esophageal necrosis, tacrolimus, liver transplant, immunosuppression, gastrointestinal toxicity

## Abstract

Acute esophageal necrosis, or “black esophagus,” is a rare but severe condition characterized by circumferential black necrotized esophageal mucosa with an abrupt transition at the gastroesophageal junction. The “two-hit” hypothesis suggests that an initial ischemic insult predisposes the esophageal mucosa to further injury from luminal acid exposure or medications. We present the first documented case of tacrolimus-induced acute esophageal necrosis without preceding hemodynamic instability in a liver transplant recipient, which resolved with discontinuation of tacrolimus and transition to cyclosporin.

## INTRODUCTION

Acute esophageal necrosis (AEN), or “black esophagus,” is a rare but severe condition characterized by circumferential black necrotized esophageal mucosa with an abrupt transition at the gastroesophageal junction.^1^ The pathogenesis follows a “two-hit” hypothesis, where an initial ischemic insult due to low perfusion, often in setting of systemic hypotension, predisposes the esophageal mucosa to severe topical injury from luminal acid exposure, infections, medications, and other etiologies.^2,3^

Various medications, including antibiotics, nonsteroidal anti-inflammatory drugs, antihypertensives, and antipsychotics, have been implicated as potential triggers. In addition, chronic medical conditions such as diabetes mellitus, atherosclerotic arterial disease, malnutrition, and chronic kidney disease may confer an increased risk of AEN.^4–6^

Tacrolimus (TAC), a calcineurin inhibitor, is widely used for immunosuppression in transplant recipients. While the association between TAC and AEN has been previously reported in a renal transplant recipient,^7^ and its link to gastric and colonic ulcers has been described in 2 cardiac transplant recipients,^8,9^ we report the first documented case of TAC-induced AEN in a liver transplant recipient.

## CASE REPORT

A 54-year-old man with a history of insulin-dependent diabetes was initiated on TAC (5 mg twice daily) and prednisone (20 mg daily) after orthoptic liver transplantation. His immediate post-transplant course was complicated by acute kidney injury and altered mentation suspected secondary to TAC nephrotoxicity and neurotoxicity in the setting of a supratherapeutic TAC level of 14 ng/mL (goal 10-12 ng/mL). Two weeks after transplantation, TAC was transitioned to cyclosporine (200 mg twice daily). The patient remained clinically and serologically stable on cyclosporine; however, 3 months after the transition, repeat laboratory testing revealed elevated aspartate aminotransferase to 461 U/L, alanine aminotransferase to 551 U/L, alkaline phosphatase to 386 U/L, and total bilirubin of 0.8 mg/dL concerning for allograft rejection. Cyclosporine was discontinued, and TAC was reinitiated (starting at 5 mg twice daily and titrated to a target trough level of 8–10 ng/mL), resulting in rapid normalization of liver enzyme levels.

Two months later, the patient presented with 2 weeks of nausea, hematochezia, odynophagia, and early satiety. He was hemodynamically stable, and patient appeared euvolemic on examination. Repeat laboratory tests noted normal aspartate aminotransferase, alanine aminotransferase, alkaline phosphatase, and bilirubin with a hemoglobin of 11.7 g/dL and TAC level of 7.0 ng/mL (goal 8-10 ng/mL).

**Figure 1.**
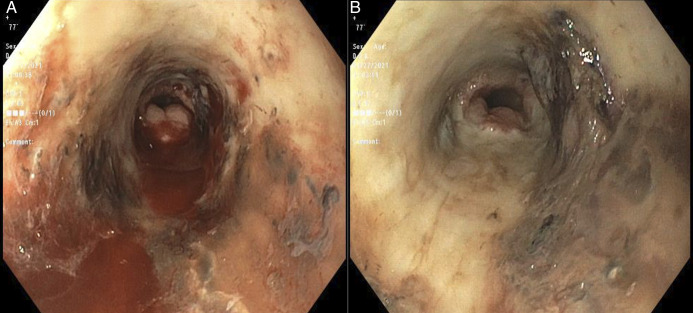
Endoscopic finding of acute esophageal necrosis. Endoscopic view of the mid-esophagus (A) showing circumferential erythema, friability, necrosis, and active bleeding extending caudally to the distal esophagus (B) with sharp demarcation at the gastroesophageal junction.

A contrast-enhanced chest computed tomography revealed diffuse mid-to-distal esophageal thickening. Subsequent esophagogastroduodenoscopy demonstrated diffuse circumferential erosive esophageal mucosa with friability, black necrosis, and mucosal ulcerations, sparing of the gastroesophageal junction (Figure 1). Histopathology confirmed mucosal ulceration with acute and chronic inflammation, and necrosis without evidence of viral, fungal, or bacterial infection.

In the absence of alternative etiologies, he was diagnosed with AEN suspected secondary to TAC; thus, tacrolimus was discontinued, and cyclosporine was reintroduced. Supportive management was initiated, including administration of intravenous fluids, high-dose proton pump inhibitors, and nil per os status, leading to gradual symptomatic improvement over 1 week.

**Figure 2.**
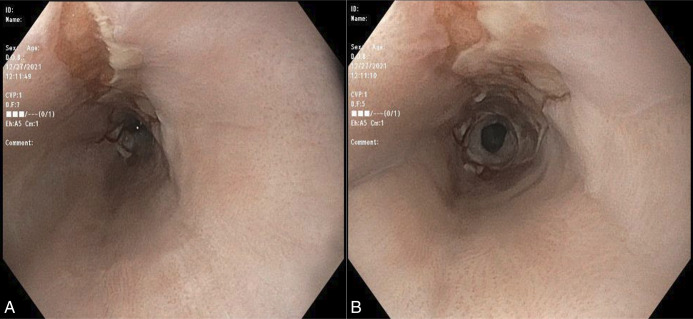
Endoscopic evolution of acute esophageal necrosis. Interval endoscopic assessment 3 months later with (A) residual longitudinal ulceration and (B) a distal esophageal stricture.

At 3-month follow-up, the patient endorsed mild dysphagia to solids. Repeat esophagogastroduodenoscopy showed resolution of necrotizing esophagitis with residual ulceration at the middle to distal esophagus and a new benign 8-mm distal esophageal stricture, successfully dilated to 12 mm via a Savary 10 mm through-the-scope balloon (Figure 2). The patient remained symptom-free at 1-year follow-up.

## DISCUSSION

AEN is a rare clinicopathologic entity with a reported prevalence ranging from 0.01% to 0.28% upon diagnostic endoscopic.^10^ Due to its rarity, there are no current standardized management guidelines; however, it carries an overall mortality rate of approximately 30%, largely due to life-threatening complications such as esophageal perforation (∼7%) and uncontrolled esophageal bleeding.^5,6^

Tacrolimus is the primary agent used for immunosuppression in solid organ transplant recipients. Its side effect profile includes nephrotoxicity, neurotoxicity, and gastrointestinal toxicity, such as colitis.^11^ Although AEN is not a well-established association, case reports have suggested a possible association between TAC use and AEN in renal transplant recipients.^7^ The exact mechanism of gastrointestinal injury remains unclear but is believed to involve crypt abnormalities, mucosal destruction, and apoptosis. A retrospective review of colonic samples from 20 patients receiving TAC monotherapy identified no necrosis but neutrophilic cryptitis (60%), apoptotic crypt cells (55%), and crypt destruction (35%), and more than half of these patients had endoscopic colitis, reflecting possible drug-induced immune dysregulation.^12^ Notably, CNIs can also cause arterial endothelial impairment, disrupting the balance between vasodilators and vasoconstrictors, and promoting vascular inflammation via toll-like receptor-4 signaling, as seen in TAC-associated cerebral vasoconstriction syndrome.^13–15^

**Figure 3.**
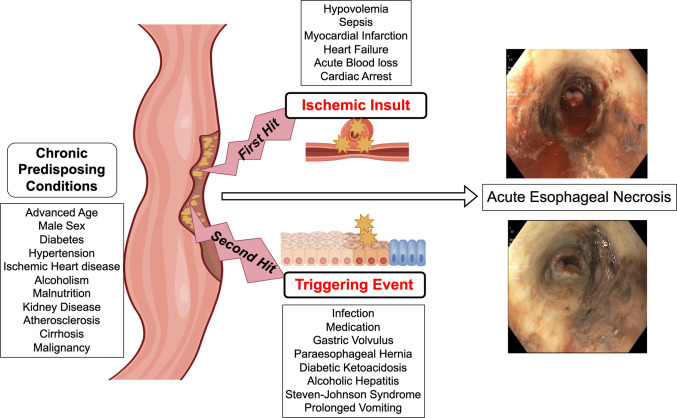
Pathophysiology of acute esophageal necrosis.

In our case, TAC may have played a central role in the “two-hit” AEN mechanism via vascular endothelial injury, vasoconstriction, and vascular inflammation, culminating in esophageal necrosis (Figure 3). The patient's risk factors, including age over 50 years, male sex, and insulin-dependent diabetes mellitus, further predisposed him to AEN, while his history of presumed TAC-induced nephrotoxicity may suggest an idiosyncratic response and heightened susceptibility to vascular injury, also increasing his risk of AEN development.^3^ This case highlights the need for clinicians understand the proposed AEN pathophysiology and recognize the interrelation among various side effects as reported in the literature.^16^

Evaluation of possible underlying etiologies should include a thorough medication review, screening for superimposed bacterial, fungal, or viral infections, and evaluation of underlying comorbid conditions. In the present case, a comprehensive infectious workup, along with histopathological evaluation of esophageal biopsy obtained during EGD—including special stains for bacterial, fungal, and viral organisms—revealed no evidence of infection. Furthermore, a detailed review of the patient's medication list identified no agents other than tacrolimus with the potential to cause esophageal necrosis. In addition to cessation of the offending agent, conservative treatment is the mainstay of management, including hydration, acid suppression therapy, total parenteral nutrition (if indicated), and sucralfate. Severe cases may surgical intervention for perforation or abscess formation.^6^ Esophageal strictures, as observed in this case, are the most common long-term complication of AEN, reported in 25% to 40% of patients, and can be successfully treated with balloon dilation, esophageal stents, or surgery.^17^ In this case, TAC discontinuation led to both clinical and endoscopic resolution of AEN.

This case of tacrolimus-induced AEN highlights a possible rare but fatal adverse effect of a commonly used immunosuppressive agent. Diagnosis of AEN requires an initial broad differential and high index of suspicion. Clinicians should recognize key triggering events, prior ischemic insults, and chronic conditions as risk factors for AEN, as early detection and discontinuation of the offending agent can significantly improve patient outcomes.

## DISCLOSURES

Author contributions: All named authors meet the International Committee of Medical Journal Editors (ICMJE) criteria for authorship for this article, take responsibility for the integrity of the work as a whole, and have given their approval for this version to be published. Abhay Dhand is the article guarantor.

Financial disclosure: None to report.

Informed consent was obtained for this case report.
